# Temporal control of gene deletion in sensory ganglia using a tamoxifen-inducible *Advillin-Cre-ERT2 *recombinase mouse

**DOI:** 10.1186/1744-8069-7-100

**Published:** 2011-12-21

**Authors:** Joanne Lau, Michael S Minett, Jing Zhao, Ulla Dennehy, Fan Wang, John N Wood, Yury D Bogdanov

**Affiliations:** 1 Molecular Nociception Group, Wolfson Institute for Biomedical Research (WIBR), Cruciform Building, University College London (UCL), London WC1E 6BT, UK; 2 Department of Cell and Developmental Biology, Wolfson Institute for Biomedical Research (WIBR), Cruciform Building, University College London (UCL), London WC1E 6BT, UK; 3 Nanaline Duke Bldg., Box 3709, Duke University Medical Center, Durham, NC 27710, USA

**Keywords:** AvCreERT2, pain and nociception, tamoxifen inducible, ROSA26 LacZ reporter, behaviour, DRG

## Abstract

**Background:**

Tissue-specific gene deletion has proved informative in the analysis of pain pathways. *Advillin *has been shown to be a pan-neuronal marker of spinal and cranial sensory ganglia. We generated BAC transgenic mice using the *Advillin *promoter to drive a tamoxifen-inducible CreERT2 recombinase construct in order to be able to delete genes in adult animals. We used a floxed stop *ROSA26LacZ *reporter mouse to examine functional Cre expression, and analysed the behaviour of mice expressing Cre recombinase.

**Results:**

We used recombineering to introduce a CreERT2 cassette in place of exon 2 of the *Advillin *gene into a BAC clone (RPCI23-424F19) containing the 5' region of the *Advillin *gene. Transgenic mice were generated using pronuclear injection. The resulting *AvCreERT2 *transgenic mice showed a highly specific expression pattern of Cre activity after tamoxifen induction. Recombinase activity was confined to sensory neurons and no expression was found in other organs. Less than 1% of neurons showed Cre expression in the absence of tamoxifen treatment. Five-day intraperitoneal treatment with tamoxifen (2 mg per day) induced Cre recombination events in ≈90% of neurons in dorsal root and cranial ganglia. Cell counts of dorsal root ganglia (DRG) from transgenic animals with or without tamoxifen treatment showed no neuronal cell loss. Sensory neurons in culture showed ≈70% induction after 3 days treatment with tamoxifen. Behavioural tests showed no differences between wildtype, *AvCreERT2 *and tamoxifen-treated animals in terms of motor function, responses to light touch and noxious pressure, thermal thresholds as well as responses to inflammatory agents.

**Conclusions:**

Our results suggest that the inducible pan-DRG *AvCreERT2 *deleter mouse strain is a useful tool for studying the role of individual genes in adult sensory neuron function. The pain phenotype of the Cre-induced animal is normal; therefore any alterations in pain processing can be unambiguously attributed to loss of the targeted gene.

## Background

Transgenic mice continue to be an important tool for the study of gene function in pain research. By 2007, over 400 papers had already been published with statistically significant behavioural pain phenotypes in transgenic knockout mice [1]. While these papers demonstrate the usefulness of transgenic knockout studies, some also reveal the drawbacks to this method. Ablation mutations introduced in the germ line may result in complications including early lethality, developmental compensatory mechanisms, and complex phenotypes when working with a pleiotropic gene [2,3]. Tissue-specific gene targeting may prevent early lethality, but temporal control would clarify the issue of compensation by other genes and negate confounding developmental roles the gene may play. Inducible tissue-specific gene targeting would thus be of great use in studies of gene function. Such a system has been previously developed using a version of the Cre-loxP system [4-7].

The Cre-loxP system can excise or invert DNA segments when flanked with 34-bp *loxP *sites. Bacteriophage-produced Cre recombinase recognizes *loxP *sites and catalyzes molecular recombination between those sites. When a tissue-specific promoter drives Cre recombinase, the DNA recombination is restricted to that tissue [8].

In a modification of the Cre-loxP system Cre recombinase is fused to a mutated ligand-binding domain of the human estrogen receptor (ER) producing CreER, which is inducible in the presence of an estrogen agonist [9]. A more efficient version of the CreER fusion protein is CreERT2, which contains 3 mutations in the human ER such that the complex is activated by the synthetic estrogen-like agonist tamoxifen, but not by endogenous estrogens [5].

While transgenic, linkage mapping and microarray studies have put forward many pain-relevant gene candidates [1,10,11], these genes are often widely expressed in both neuronal and non-neuronal tissues, making their specific function in pain pathways particularly challenging to ascertain. Peripheral sensory neuron specific gene deletion would therefore help to clarify the role of specific genes in pain pathways. To accomplish this, a peripheral sensory neuron specific promoter was used to drive CreERT2 expression. *Advillin *(also known as *Pervin*) is an actin-binding protein of gelsolin family found almost exclusively in peripheral sensory neurons [12-14] making it particularly relevant to nociception and pain research [15-17]. An *Advillin *knock-in Cre has been generated and is capable of deleting genes in all sensory neurons [18]. By placing CreERT2 under the control of the *Advillin *promoter, one can manipulate genes specifically in peripheral sensory neurons. Here, we describe the production and characterisation of *Advillin-CreERT2 *(AvCreERT2) mice in terms of expression patterns and the efficiency of recombination. AvCreERT2 and tamoxifen treatment are also shown to have no behavioural effects in various pain models.

## Results and discussion

### Generation of *AvCreERT2 *transgenic mice

**Figure 1:**
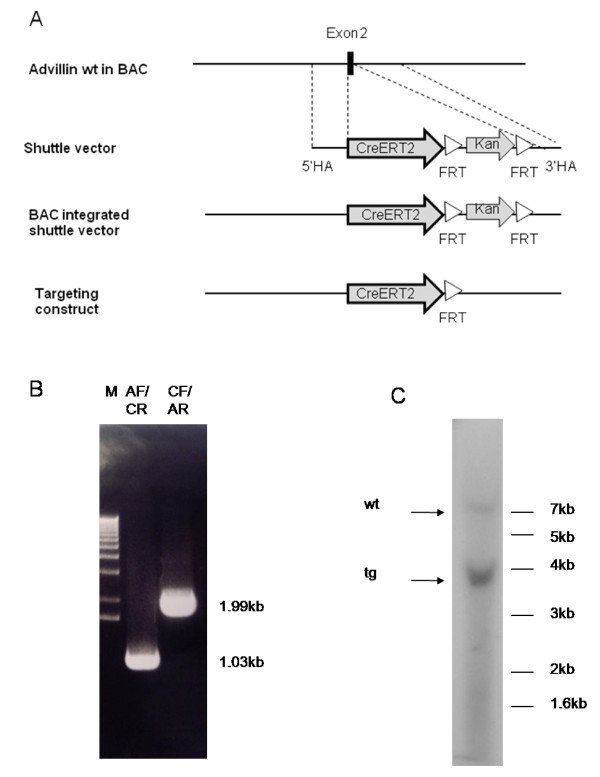
**Generation of tamoxifen inducible *Advillin Cre *mouse**. **A **- Diagram of the wt *Advillin *allele and the targeting construct. CreERT2 is inserted downstream of the initiation codon of *Advillin*. The construct also contained a *Kanamycin *selection gene that was excised from the BAC by Flp-recombination. **B **- Primer validation for screening of transgenic animals. AF - *Advillin *forward primer, AR - *Advillin *reverse primer, CF - Cre forward primer, CR - Cre reverse primer. The expected sizes of the PCR fragments are indicated. **C **- Southern blot with tail genomic DNA digested with HindIII and hybridized with the 5'homology arm probe. The bands for wildtype gene (wt) and transgene (tg) are indicated.

We generated *AvCreERT2 *transgenic mice using a construct obtained by recombineering protocol [19] (Figure 1A). BAC clone RPCI23-424F19 containing part of the *Advillin *gene was used as a template to prepare a targeting construct. The shuttle vector for recombineering was made by inserting the 5'-homology arm (0.45 kb) and the 3'-homology arm (0.3 kb) flanking exon 2 of the *Advillin *gene into a vector containing CreERT2 and a *Kanamycin *cassette. The *Kanamycin *resistance gene in the cassette was flanked by *FRT *sites (recognition sites for Flp-recombinase). In the resulting shuttle vector we fused the CreERT2 sequence with the start codon in the exon 2 of the *Advillin *gene. The completed shuttle construct was sequenced and the targeting cassette was isolated from the plasmid by AscI/PacI digest.

EL250 *E. coli *cells transformed with the BAC clone were co-transformed with the shuttle targeting construct and recombination was induced by heat-shock. The induced bacterial clones were isolated by growing them on *Kanamycin*-containing plates. The *Kanamycin *resistance cassette was removed by inducing Flp-recombinase expression by arabinose. The final construct was verified by PCR and analytical restriction digests by NotI and PacI. The resulting BAC containing the targeting cassette was used for pronucleus injection.

We obtained 8 founders verified by PCR analysis that transmitted CreERT2 to their offspring. Primers for PCR analysis are described in Materials and Methods and their verification is presented in Figure 1B. The offspring of the founders was crossed with ROSA26RLacZ mice and their various tissues were stained with X-gal following tamoxifen injections. The offspring of the founder A displayed the most profound staining in the DRG and was used for all the subsequent experiments.

To estimate the copy number of the transgenes in the offspring of founder A, we performed a Southern blot on genomic DNA from isolated from tail snips. The DNA was digested with HindIII and hybridized with the 5' homology arm probe. This probe recognizes both the wildtype and transgene variants. The resulting wildtype band was 7.4 kb and the transgene band was 3.6 kb (Figure 1C). We scanned the relative densities of the bands using ImageJ software and found that after background subtraction the value of transgene band is roughly 5 times higher than the wildtype band. Given that the wildtype band corresponds to 2 copies of *Advillin *per genome, we estimate that the offspring of founder A had about 10 copies of BAC integrated into the genome.

### Expression pattern of *AvCreERT2 *in DRG neurons

To determine whether the *Advillin *promoter driven Cre expression pattern recapitulates the expression pattern of wildtype *Advillin *we crossed *AvCreERT2 *mice with the *Rosa26LacZ *mouse strain [20]. In the *Rosa26LacZ *strain floxed polyadenylation signal blocks the expression of β-galactosidase. Introduction of Cre in a tissue-specific manner leads to the synthesis of functional β-galactosidase, which is detectable with X-gal staining [20].

**Figure 2:**
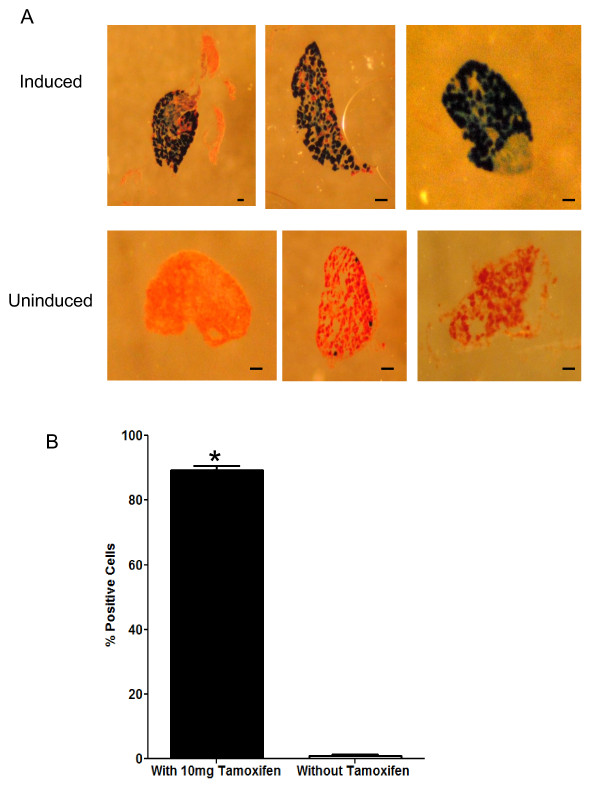
**Tamoxifen induces recombination in the DRG of adult *AvCreERT2 *mouse**. Animals were injected (ip) for 5 consecutive days (2 mg per day). **A **- X-gal staining of adult DRG neurons from induced and un-induced mice. Neutral red was used for counterstaining. **B **- Quantification of recombination events in DRG of untreated and tamoxifen-treated animals. Data are presented as mean ± SEM. Statistical analysis - unpaired T-test, p ≤ 0.01. Scale bars = 40 μm.

Tamoxifen was injected intraperitoneally (ip) for 5 consecutive days, 2 mg per day (10 mg total dose). The control animals were injected with vehicle only. Animals were euthanized and perfused with 4% paraformaldehyde. DRG were isolated and sectioned. The sections were stained with X-gal. Figure 2A shows DRG sections from tamoxifen-treated mice, where most neurons show positive blue staining whilst DRG sections from control mice show less than 1% Cre positive staining. This is probably due to spontaneous Cre activation. Quantification of results from three independent experiments is shown in Figure 2B. DRG sections from tamoxifen injected mice show on average 89% of Cre positive staining whereas control animals show 0.95% Cre positive blue staining. It is important to note that in all DRG sections from tamoxifen-treated mice, the percentage of cells with Cre positive staining was never lower than 82% and the maximum recorded staining was 99.2%. These results demonstrate that *AvCreERT2 *transgenic mice are a useful pan-DRG inducible deleter strain.

**Figure 3:**
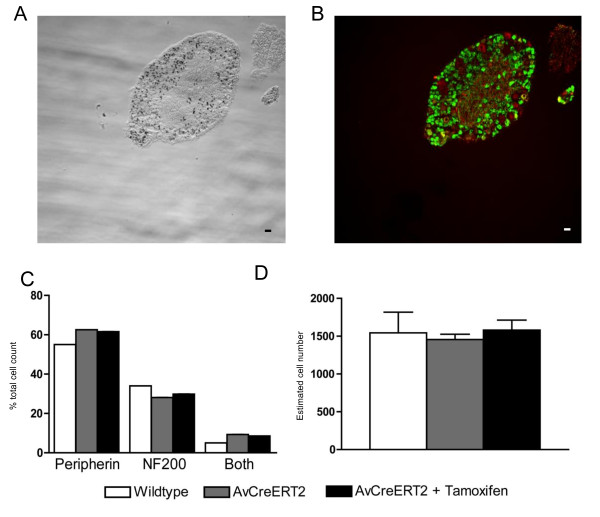
**Co-localization of X-gal staining with peripherin and NF200 positive neurons in the dorsal root ganglia of the adult AvCreERT2 mice**. **A **- X-gal (transmitted light) and **B**. - Anti-peripherin (green) and anti-NF200 staining (red). **C **- Tamoxifen treatment does not alter the composition of DRG. White bars - wt, Grey bars - uninjected AvCreERT2 animals, Black bars - AvCreERT2 animals injected with tamoxifen (2 mg per day, 5 days). **D **- Quantification of the total number of neurons per DRG of wildtype and AvCreERT2 mice (untreated and injected with tamoxifen). Data are presented as mean ± SEM. Scale bars = 40 μm.

To assess the types of sensory neurons that express the transgene we labelled DRG sections with antibodies against peripherin (Figure 3B in green) and neurofilament-200 (Figure 3B in red). The same sections were stained with X-gal to characterize Cre-recombinase activity (Figure 3A). The number of peripherin and neurofilament-200 expressing neurons in tamoxifen-treated *AvCreERT2/Rosa26LacZ *mice correlates to historical data from wildtype mice [21] (Figure 3C). This indicates that transgene insertion and/or tamoxifen treatment does not change the relative proportion of sensory neurons. We further investigated the actual numbers of sensory neurons per DRG and compared it to historical data from wildtype mice (Figure 3D). No differences were found in the actual numbers of neurons in the DRG of wildtype animals, untreated and tamoxifen-treated *AvCreERT2 *transgenic animals.

Our data suggest that the insertion of the *AvCreERT2 *transgene and tamoxifen treatment does not alter the number or the relative proportion of sensory neurons in DRG.

We also performed X-gal staining on a variety of tissue sections from tamoxifen-treated mice (results not shown). These tissues included brain, liver, lungs, kidney, olfactory epithelium, adrenal glands, skin etc. No positive Cre staining was observed. These results prove that *AvCreERT2 *transgene expression recapitulates the previously reported expression pattern of wildtype *Advillin *[13,22].

### Tamoxifen induces Cre-expression during embryonic development

**Figure 4:**
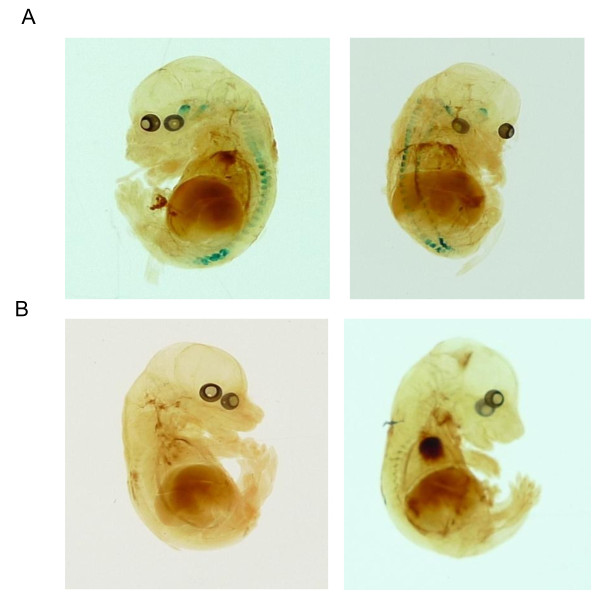
**Tamoxifen induces Cre-expression during embryonic development**. **A **- E18.5 AvCreERT2-positive embryos from tamoxifen treated pregnant females (2 mg per day, 5 days). **B **- E18.5 AvCreERT2-positive embryos from vehicle treated pregnant females.

To assess the possibility of Cre induction during embryonic development we set up timed matings of AvCreERT2/ROSA26LacZ mice. It has been shown that expression of *Advillin *starts at day E11.5 in trigeminal ganglia. In DRG neurons *Advillin *staining is clearly visible at day E12.5 [22]. We started to inject pregnant mice at day E12.5 for 5 consecutive days with 2 mg of tamoxifen per day. Embryos were harvested at E18.5 and stained with X-gal. Embryos from tamoxifen-treated mice displayed clear positive staining in DRG and trigeminal ganglia (Figure 4). All other organs showed no positive Cre staining. Untreated embryos displayed no positive Cre staining. These data are in agreement with the previously published data on the *Advillin *gene expression [22].

### 4-Hydroxytamoxifen induces Cre-expression in cell culture

**Figure 5:**
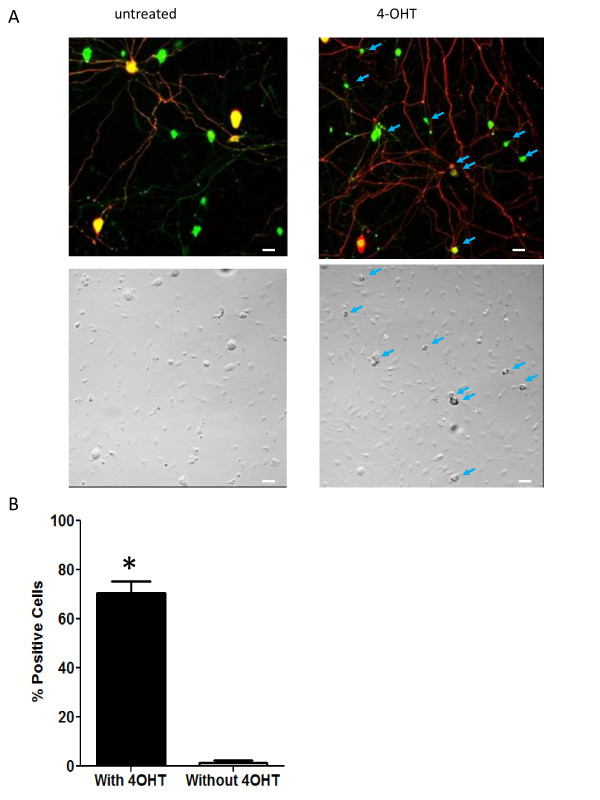
**4-OHT induces Cre-expression in cell culture of neurons from dorsal root ganglia of AvCreERT2 mice**. **A **- X-Gal, anti-peripherin (green) and anti-NF200 (red) staining of 4-OHT treated and uninduced cell cultures from DRG. Arrows indicate X-gal stained neurons. Note the punctuate nature of X-gal staining. **B **- Quantification of recombination events in untreated and 4-OHT treated cultures of neurons from DRGs. Data are presented as mean ± SEM. Statistical analysis - unpaired T-test, p ≤ 0.01. Scale bars = 40 μm.

We also investigated the ability of tamoxifen to induce Cre recombination in cultured DRG neurons from *AvCreERT2/ROSA26LacZ *mice. Cell cultures were treated with 4-Hydroxytamoxifen (4-OHT) for 3 consecutive days. The cell cultures were treated with 1 μM 4-OHT on the first day and with 2 μM of 4-OHT on the two subsequent days (Figure 5A). We also tried 10 μM 4-OHT treatment but found it to be toxic. The neurons were fixed and stained with X-gal and antibodies against peripherin and neurofilament-200. The results were quantified and are presented in Figure 5B. Tamoxifen induces Cre recombination in about 70% of cultured DRG neurons. Virtually no Cre positive staining was observed in the untreated cultures.

### *AvCreERT2 *expressing mice and tamoxifen-treated show normal acute and inflammatory pain behaviour

**Figure 6:**
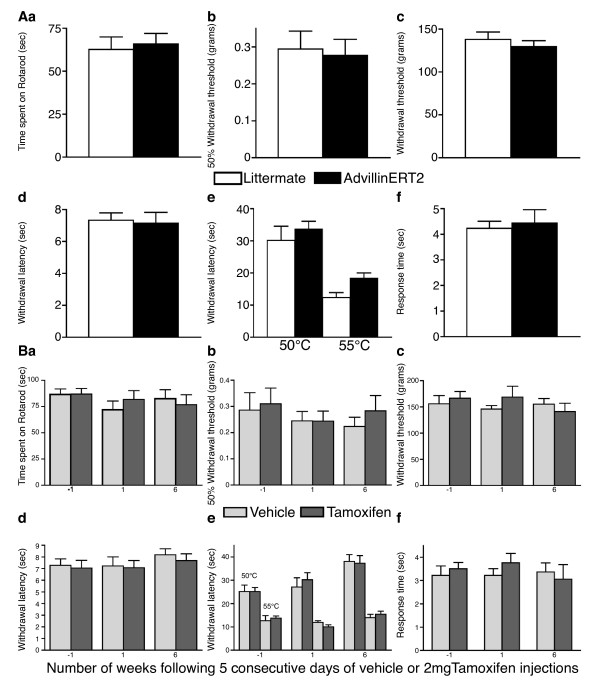
**AvCreERT2 expression or tamoxifen treatment (10 mg) does not affect acute nociceptive responses**. **A **- Behavioural responses of *AvCreERT2 *and littermate mice to Aa) Rotarod (AvCreERT2 N = 13, littermate N = 11), Ab) von Frey (AvCreERT2 N = 13, littermate N = 11), Ac) Randall-Selitto (AvCreERT2 N = 8, littermate N = 8), Ad) Hargreaves' (AvCreERT2 N = 13, littermate N = 11), Ae) (AvCreERT2 N = 7, littermate N = 8) Af) Acetone (AvCreERT2 N = 8, littermate N = 8). No significant difference was found (t-test). Data are expressed as mean ± SEM. **B **- C57BL/6 mice received a five-day course of tamoxifen (2 mg/0.2 ml per day), or vehicle (0.2 ml per day). Responses to Ba) Rotarod (tamoxifen N = 12, vehicle N = 11) Bb) von Frey (tamoxifen N = 12, vehicle N = 11). Bc) Randall-Selitto (tamoxifen N = 6, vehicle N = 5) Bd) Hargreaves' (tamoxifen N = 12, vehicle N = 11) Be) 50 & 55°C Hot plate (tamoxifen N = 12, vehicle N = 11) Bf) Acetone (tamoxifen N = 12, vehicle N = 11). Animals were examined 7 days prior to tamoxifen treatment then 7 and 56 days after. No significant differences were found (Two-way repeated measures ANOVA). Data are expressed as mean ± SEM.

**Figure 7:**
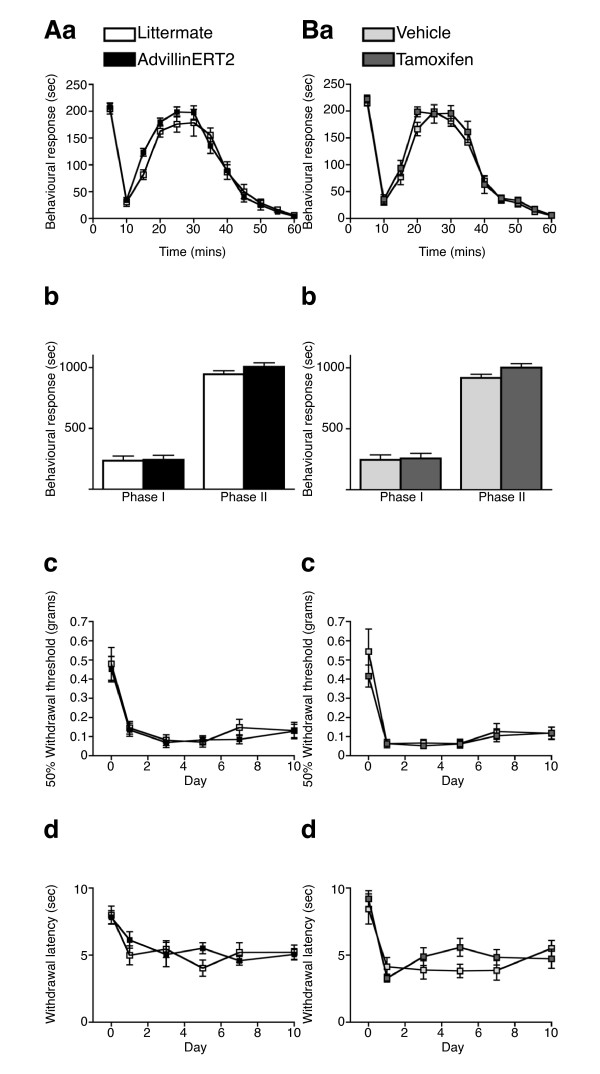
**AvCreERT2 expression or tamoxifen (10 mg) treatment does not affect inflammatory nociceptive responses**. **A **- Behavioural responses of AvCreERT2 and littermate mice to inflammatory pain tests Aa) Pain behaviour of AvCreERT2 (N = 6) & littermate (N = 6) mice after intraplantar injection of 20 μl of 5% formalin. Ab) Time spent licking/biting the injected hindpaw in phase I (1-10 min) and phase II (10-60 min) (AvCreERT2 N = 6, littermate N = 6), Ac) Mechanical allodynia following intraplantar injection of 20 μl of CFA (AvCreERT2 N = 7, littermate N = 6), Ad) Thermal hyperalgesia after intraplantar injection of 20 μl of CFA (AvCreERT2 N = 7, littermate N = 6). No significant difference was found (Two-way repeated measures ANOVA). Data are expressed as mean ± SEM. **B **- Behavioural responses of C57BL/6 mice injected with a five-day course of tamoxifen (2 mg/0.2 ml per day), or vehicle (0.2 ml per day) to inflammatory pain tests. Ba) Pain behavior of tamoxifen-treated (N = 6) & vehicle-treated (N = 6) mice after intraplantar injection of 20 μl of 5% formalin. Bb) Time spent licking/biting the injected hindpaw in phase I (1-10 min) and phase II (10 -60 min) (tamoxifen N = 6, vehicle N = 6), Bc) Mechanical allodynia following intraplantar injection of 20 μl of CFA (tamoxifen N = 6, vehicle N = 6), Bd) Thermal hyperalgesia after intraplantar injection of 20 μl of CFA (tamoxifen N = 6, vehicle N = 6). Animals were examined 7 days following tamoxifen/vehicle treatment. No significant differences were found (Two-way repeated measures ANOVA). Data are expressed as mean ± SEM.

Motor function was assessed using the Rotarod test. The mean time spent on the apparatus was not significantly different for *AvCreERT2 *mice (Figure 6Aa) or following tamoxifen treatment (Figure 6Ba). Mechanical thresholds were assessed using the von Frey and Randall-Selitto tests. The mean response thresholds for both tests were not significantly different for *AvCreERT2 *mice (Figure 6Ab&6Ac) or following tamoxifen treatment (Figure 6Bb&6Bc). This suggest both responses to light touch and noxious pressure are unaffected by *AvCreERT2 *or tamoxifen. Thermal thresholds were assessed using the Hargreaves', hot plate (50°C & 55°C) and acetone test. The mean withdrawal latencies to both the Hargreaves' and hot plate tests were not significantly different for *AvCreERT2 *mice (Figure 6Ad&6Ae) or following tamoxifen treatment (Figure 6Bd&6Be). This suggests that both spinal reflexes and supra-spinal processing of noxious heat are unaffected by *AvCreERT2 *or tamoxifen [23]. Similarly the mean response times to the acetone test was not significantly different for *AvCreERT2 *mice (Figure 6Af) or following tamoxifen treatment (Figure 6Bf). This suggests that processing of noxious cooling stimuli are unaffected by *AvCreERT2 *or tamoxifen. Finally, the behavioural responses to both 5% formalin and CFA intraplantar injection were not significantly different for *AvCreERT2 *mice or following tamoxifen treatment (Figure 7).

## Conclusions

We generated a transgenic *AvCreERT2 *mouse line that displays functional expression of Cre recombinase after tamoxifen induction. We described molecular and behavioural characterization of this line. The *Advillin *promoter drives the expression of Cre recombinase with the same characteristics as the wildtype *Advillin *gene. The expression of this gene is specific to sensory neurones making it very useful for creating pan-DRG deleter mouse strains.

The results described in this paper show that we are able to induce Cre expression in 89% of DRG neurons in vivo (5 injections, 2 mg per day) and in 70% of neurons in vitro (4-OHT treatment for 3 days). We compared the proportion of anti-peripherin and anti-neuropfilament-200 stained neurons in *AvCreERT2 *DRG sections with wildtype DRG sections and found no differences. We also found no differences in the actual numbers of DRG neurons in wildtype animals, and untreated and tamoxifen treated *AvCreERT2 *transgenic animals.

These results suggest that the neuronal composition of *AvCreERT2 *DRG mice is the same as wildtype mice. Behavioural studies of wildtype, untreated and tamoxifen treated and *AvCreERT2 *mice show that motor function, responses to light touch and noxious pressure, as well as thermal thresholds do not differ significantly between these animals.

Our results suggest that we generated an inducible pan-DRG deleter mouse strain that can be used to study the role of individual genes in pain transduction in vivo and their role in cell excitability in vitro. Thus this mouse strain may provide mechanistic insights that identify new therapeutic pain targets.

## Methods

### Animals

All tests were approved by the United Kingdom Home Office Animals (Scientific Procedures) Act 1986. Experiments were conducted using both male and female littermate mice, all of which were at least 6 weeks old when tested. The same observer performed all experiments and was blind to the genotype of the animals.

### Generation of transgenic construct

To create the construct for production of the transgenic *AvCreERT2 *mouse line we used a recombineering technique [19]. The CreERT2 containing plasmid which also contained a *FRT *flanked *Kanamycin *selection cassette was a gift from Prof Chambon [9].

Briefly, the start codon of *Advillin *is located in exon 2 of the gene. The 5' homologous arm of the construct (0.45 kb) was amplified from a BAC containing exon 2 of the *Advillin *gene and cloned into the AscI/SalI restriction sites of the CreERT2 vector. The 3'homologous arm (0.3 kb) was amplified from the same BAC and cloned into the PacI/EagI restriction sites of the CreERT2 vector. The completed shuttle construct was sequenced and the targeting cassette was isolated from the plasmid by AscI/PacI digest.

EL250 *E. coli *cells were transformed with the BAC containing the *Advillin *gene. Isolated BAC DNA from the individual clones was checked by restriction analysis followed by pulse-field gel electrophoresis. Only the clones displaying the correct pattern of the restriction fragments were used for the recombineering procedure. The correct bacterial clones were co-transformed by shuttle construct and recombination was induced by incubating the bacteria at 42°C for 15 minutes. The induced bacterial clones were isolated by growing them on *Kanamycin *containing plates. The *Kanamycin *resistance cassette was removed by inducing Flp-recombinase expression by arabinose. The final construct was verified by PCR and analytical restriction digests using NotI and PacI.

### Transgenic mouse production and screening

The resulting BAC was isolated, digested by Not I, gel purified and used for the oocyte microinjection. The founders were screened by PCR and Southern blot. The primers for PCR are as follows:

AdF:GACAGATTATCTGCAATCTCTCTAAG, AdR:AGAGCACAGAGCCACCCTCGAGAC, CREF:GGCCTGGTCTGGCCACTCTGCCAG, CRER:GTTCCTGATGTCCTGGCATCTGTC.

The products of PCR reactions are AdF/AdR - 0.95 kb(wt) and 3.39(tg), AdF/CreR - 1.027 kb and CreF/AdR - 1.99 kb. The products were resolved on 1% agarose gel, stained with ethidium bromide and photographed.

### Southern blot analysis

Genomic DNA was extracted from tail snips [24]. The 5' probe which recognizes both the wildtype gene and transgene was amplified from BAC using the following primers Avil5HS: GACGGCGCGCCCTCAGGAATATG TGTTGCCTTTC; Avil5HAS: TCTGTCGACCATAGTGGCTGTCTTCCTGGAAC.

Southern blot analysis with HindIII digested genomic DNA produced a 7.4 kb wildtype band and a 3.6 kb transgene band (Figure 1C).

### Tamoxifen treatment

Tamoxifen (Sigma T5648) and 4-OHT (Sigma H7904) solutions were prepared according to a previously described protocol [9]. Eight week old AvCreERT2/ROSA26LacZ mice were injected with 2 mg of tamoxifen (ip) daily for 5 consecutive days. Two days after the last injection mice were euthanized and DRG were collected for further analysis. To characterize Cre induction during embryonic development, pregnant *AvCreERT2/ROSA26LacZ *mice were injected with either 2 mg of tamoxifen or vehicle daily for 5 consecutive days starting at day E12.5. On day E18.5 the mice were euthanized and the embryos were collected for further analysis. All embryos were genotyped.

Cultured DRG neurons were treated with 4-OHT for 3 days (1 μM on the first day and 2 μM the following two days). The neurons were fixed and analyzed the day after the final treatment.

### DRG cell culture

*AvCreERT2/ROSA26LacZ *animals were killed by inhalation of a rising concentration of CO_2 _followed by cervical dislocation, and 30-40 DRG were dissected from each. Ganglia were digested in collagenase (Type XI, 0.6 mg/ml, Sigma), dispase (3.0 mg/ml, Sigma), and glucose (1.8 mg/ml) in Ca^2^+, Mg^2^+ free PBS for 40 min prior to mechnical trituration. Cells were then resupended in Dulbecco's modified Eagle's medium (Gibco) containing 10% fetal bovine serum (Gibco), 10,000 i.u./ml penicillin-streptomycin (Gibco), and 100 ng/ml nerve growth factor (Sigma), and plated on 13-mm cover slips coated with poly-L-lysine.

### Immunohistochemistry

The staining was performed as previously described [25]. Briefly, following the tamoxifen treatment, the DRGs were isolated and immediately frozen in OCT (O.C.T. Compounds, BDH) on dry ice. 12 μm cryosections were dried at room temperature for 30 min and then fixed with 4% PBS-buffered paraformaldehyde solution for 10 min on ice. After three washes in PBS, sections were incubated for 30 min in 10% goat serum diluted in PBS containing 0.3% Triton X-100 (PBST), and for 1 h at room temperature in a 1:500 dilution of an anti peripherin monoclonal antibody (P5117, Sigma) and 1:200 dilution of an anti N-200 polyclonal antibody (N4142, Sigma). Following three washes in PBST, the sections were incubated for 1 h in a 1:500 dilution of Alexa Fluor 488 goat anti-mouse IgG (A-11017, Molecular Probes) and 1:1,000 dilution of Alexa Fluor594 goat anti-rabbit IgG (A-11037, Molecular Probes). After three washes in PBST, the sections were mounted in CITIFlour solution and analysed using a fluorescent microscope.

### X-gal staining

After fixation with 4% PBS-buffered paraformaldehyde or after immunohistochemistry, DRG sections were washed three times with PBS and then incubated overnight in X-gal solution at 35°C. For cultured DRG neurons, the slides were fixed with 2% PBS-buffered formaldehyde containing 0.25% glutaraldehyde for 15 min at room temperature, and then stained with X-gal solution at 35°C overnight. Sections or slides (cultured DRG neurons) were counterstained with 1% neutral red, dehydrated with ethanol, and cleared with Histo-ClearII and mounted with DePex mounting medium (BDH). The X-gal staining in freshly frozen sections is usually punctate in appearance, but homogeneous in perfused DRG sections and in cultured DRG neurons.

The staining of the E18.5 embryos was performed as follows. The embryos were isolated from pregnant females killed by CO2 inhalation and cervical dislocation. The embryos were placed immediately into 1:40 dilution of sodium pentobarbitone in PBS for 5 min at room temperature. This was followed by 3 washes with PBS and the embryos were skinned to allow better penetration of staining solutions. Tail biopsies were taken for genotyping. The embryos were fixed at room temperature for 2 hours, washed and left over night in the LacZ staining solution at room temperature. The next day embryos were washed with PBS and were cleared by incubating for 24 hours at 4°C in the following solutions: 70% ethanol, 100% methanol, fresh 100% methanol and benzyl benzoate:benzyl alcohol (2:1). Cleared embryos were photographed and analyzed.

### Behavioural studies

The motor coordination of transgenic and wildtype mice was assessed using the Rotarod test [26] before testing nociceptive responses.

#### Thermal nociception

Thermal nociceptive thresholds were measured using the paw-withdrawal latency according to the method described by [27], with minor modifications. As well as the hot-plate test (50 & 55°C) originally described by [28] and later modified by [29]. Behavioural responses to cooling (approx. 10-15°C) were assessed using the acetone test, as described by [30].

#### Mechanical nociception

Mechanical nociceptive thresholds were measured using a modified version of the Randall-Selitto test [31] that applies pressure to the tail via a 3 mm^2 ^blunt probe [32]. Touch perception thresholds were measured using the up-down method for obtaining the 50% threshold using von Frey hairs as described by [33].

## List of abbreviations used

DRG: dorsal root ganglion; AvCreERT2: Advillin-CreERT2; BAC: bacterial artificial chromosome; 4-OHT: 4-hydroxytamoxifen.

## Competing interests

The authors declare that they have no competing interests.

## Authors' contributions

JNW and FW conceived the study; YB and JZ generated the construct and UD made the mouse which was analysed by YB, JL, MSM and JZ. All authors read and approved the manuscript.
